# Evaluation of clinical outcomes after partial horizontal laryngectomy^☆^^☆☆^^☆☆☆^

**DOI:** 10.1016/j.bjorl.2025.101614

**Published:** 2025-05-29

**Authors:** Daniel Abreu Rocha, Gustavo Fernandes de Alvarenga, Daniel Marin Ramos, Leandro Luongo de Matos, Rogerio Aparecido Dedivitis, Marco Aurélio Vamondes Kulcsar, Claudio Roberto Cernea

**Affiliations:** a Universidade de São Paulo (USP), Faculdade de Medicina (FM), Hospital das Clínicas (HC), São Paulo, SP, Brazil; b Universidade de São Paulo (USP), Faculdade de Medicina (FM), Instituto do Câncer de São Paulo (ICESP), São Paulo, SP, Brazil

**Keywords:** Laryngeal neoplasms, Laryngectomy, Larynx, Open partial laryngectomy

## Abstract

**Objective:**

Evaluate the oncological and functional outcomes of patients submitted to HPL for the treatment of LSCC.

**Methods:**

A retrospective descriptive study of patients submitted to HPL, performed at a cancer referral center, between January 2011 and December 2017.

**Results:**

We evaluated 37 patients. The major pathological staging of the primary tumor was pT3 (35.1%), followed by pT2 (32.4%). Five patients required adjuvant radiotherapy; 62.2% of the patients were decannulated by the end of the treatment; 10.8% weren’t decannulated; 8.1% underwent a retracheostomy, and 18.9% had total laryngectomy. From the patients submitted to total laryngectomy, 3 cases were due to rehabilitation failure, 2 due to recurrence and 2 cases due to postoperative suture dehiscence. About 89% of the patients resumed oral feeding following the procedure; 86.4% didn’t present disease recurrence; 31 patients survived without disease; 3 died from disease-related causes, and 3 from unrelated causes.

**Conclusion:**

HPL is an alternative procedure to total laryngectomy, presenting adequate rates of local control and overall survival while also maintaining laryngeal function. The main challenge to HPL remains achieving an ideal selection of patients.

**Level of evidence:**

Level IV.

## Highlights

• Supracricoid horizontal laryngectomy provides a high rate of deccanulation and swallowing.
• It was commonest surgical procedure up to T3 supraglottic cancer.
• 14% of patients required total laryngectomy for poor functional status.

## Introduction

Laryngeal Squamous Cell Carcinoma (LSCC) accounts for about 2% of malignant tumors.^1^ In Brazil, it was estimated 6,390 new cases in men and 1,280 in women for each year of the 2018–2019 biennium.^2^ The most common subsite is the glottis region, which accounts for approximately half of the cases.^1^

Currently, the preferred treatment for early laryngeal cancer is unimodal, either surgery or radiotherapy, while advanced tumors usually undergo multimodal treatment.^3^, ^4^ For initial, intermediate and selected cases of advanced tumors, organ preservation treatments are preferred to total laryngectomy. The surgical approach, whether open or endoscopic, has shown better oncologic outcomes compared to radiotherapy.^1^, ^4^

Suprachicoid Horizontal Partial Laryngectomy (SHPL) is an open surgical procedure indicated for the treatment of selected cases of laryngeal tumors, especially early or moderately advanced glottic and supraglottic tumors. It is as an alternative treatment that offers adequate oncologic control comparable to total laryngectomy. Initially described in 1959 by Majer and Rider^5^ and modified in 1974 by Piquet et al.,^6^ SHPL consists of the complete removal of thyroid cartilage, vocal folds (false and true), paraglottic and pre-epiglottic spaces and epiglottis, associated with the preservation of cricoid cartilage, hyoid bone and at least one mobile and functional cricoarytenoid unit. Reconstruction may be performed with fixation of the remaining epiglottis and hyoid to the cricoid cartilage (cricohyoidoepiglotopexy) or fixation of the hyoid bone to the cricoid (cricohyoidopexy) according to the extent of resection.^7^, ^8^

Unlike total laryngectomy, SHPL allows voice preservation and swallowing without the need for definitive tracheostomy. It is also useful as salvage surgery in relapsed patients after radiotherapy or previous endoscopic surgery.^9^, ^10^, ^11^ Thus, SHPL, as well as other open partial laryngectomy techniques, plays an important role in the treatment of laryngeal cancer with the purpose of organ preservation, and can be used for both intermediate cases and surgical salvage of radioresistant cancers or tumor recurrence.^12^, ^13^, ^14^, ^15^, ^16^

This study is a retrospective evaluation of the oncological and functional outcomes of patients undergoing supracricoid partial laryngectomy for treatment of laryngeal squamous cell carcinoma in a reference institution for cancer treatment.

## Methods

All patients submitted to SHPL were selected at the “Instituto do Câncer do Estado de São Paulo” (ICESP), from January 2011 to December 2017.

Data on demographic characteristics, adjuvant treatment, swallowing rehabilitation, decannulation time, disease recurrence, recurrence treatment and mortality were retrospectively obtained. Overall survival, local control, and functional swallowing rehabilitation were used to evaluate clinical outcomes after partial supracricoid laryngectomy.

We performed a descriptive analysis of the data, reporting the rates found of the parameters analyzed relatively or absolutely, according to their frequency.

## Results

In our institution, from January 2011 to December 2017, 37 patients underwent partial horizontal laryngectomy. Of these, 35 (94.6%) were male and 2 (5.4%) were female. The average age of the population studied was 62.08-years.

The patients were staged according to the 8th edition of TNM,^17^ using direct laryngoscopy performed in the office and radiological examination (computed tomography or magnetic resonance) and assessing the pathological staging after resection (Table 1).

**Table 1:** Clinical and pathological staging of patients according to the 8th edition of TNM.^17^

	cTNM	pTNM
T1a	2.7%	2.7%
T1b	18.9%	21.6%
T2	40.5%	32.4%
T3	37.8%	35.1%
T4a	0	5.4%

Regarding the surgical procedure, 36 patients (97.3%) underwent supracricoid partial open laryngectomy with cricohypoid epiglottopexy (OPHL type IIa by the classification proposed by the European Society of Laringology)^18^ and 1 patient (2.7%) underwent supratracheal horizontal partial laryngectomy (OPHL type III). Of the patients undergoing supracricoid laryngectomy, 15 (41.6%) had arytenoid resection (OPHL type IIa + ARY), while 21 patients (58.4%) had no arytenoid sacrifice (Table 2).

**Table 2:** Types of Treatment by Horizontal Partial Laryngectomy (HPL) Subtype.

	Supracricoid HPL with CHEP without arytenoid sacrifice	Supracricoid HPL with CHEP and arytenoid sacrifice	Supratracheal HPL
First choice	16	15	1
Rescue after previous endoscopic surgery	4	0	0
Rescue after radiotherapy	1	0	0

Most patients in our series had horizontal partial laryngectomy with initial treatment (86.5%). Only 5 patients (13.5%) were indicated as surgical salvage, and in four cases the patients had undergone endoscopic surgical treatment and in one case, the initial treatment had been radiotherapy (Table 2).

After surgical treatment with supracricoid partial laryngectomy, most patients in our institution had no indication for adjuvant treatment (83.8%).

Among HPL patients, 23 (62.2%) were successfully decannulated at the end of treatment, 3 (8.1%) had to undergo a new tracheostomy, 4 (10.8%) were not decannulated, and 7 (18.9%) underwent laryngectomy totalization. Among the retracheostomized patients, the causes were recurrence, respiratory failure due to mucosal redundancy and subglottic stenosis (Table 3).

**Table 3:** Functional outcomes in Horizontal Partial Laryngectomies (HPL).

**Food Rehabilitation**
Returned exclusive oral feeding: 33 (89.2%)
Alternative food tract: 4 (10.8%)
**Respiratory rehabilitation**
Successfully decannulated: 23 (62.2%)
Decannulated, but had to undergo re-tracheostomy: 3 (8.1%)
Relapse: 1 (33%)
Ins. Respiratory Mucosal Redundancy: 1 (33%)
Supraglottic Stenosis: 1 (33%)
Decannulation failure: 4 (10.8%)
Supraglottic Stenosis: 2 (50%)
Mucous Redundancy: 1 (25%)
Repeat bronchial aspiration: 1 (25%)
Subject to HPL totaling: 7 (18.9%)
Relapse: 2 (28.6%)
CHEP dehiscence: 3 (42.9%)
Repeat bronchial aspiration: 2 (28.6%)

The causes of decannulation failure were subglottic stenosis in two cases, mucosal redundancy in one case, bronchoaspiration in one case.

Regarding swallowing rehabilitation, 33 patients (89.2%) resumed oral feeding, 3 patients (8.1%) had no effective swallowing and 1 patient (2.7%) had to resume alternative feeding due to relapse of laryngeal disease (Table 3). Regarding swallowing classification by the National Outcomes Measurement System scale of the American Speech-Language-Hearing Association, 1 level I patient (2.7%), 2 level III patients (5.4%), 9 level V (24.3%), 12 level VI patients (32.4%) and 13 level VII patients (35.1%). The ASHA NOMS scale serves as a tool to determine the type of diet the patient may have access to and the type of follow-up that should be employed in each case.

Regarding totalization indications, 2 cases (28.6%) were due to tumor recurrence, 2 were due to surgical wound dehiscence and 3 cases (42.9%) were due to failed rehabilitation with repeated bronchoaspiration (Table 3). The average time for indication of total laryngectomy after partial laryngectomy was 17.14 months.

In our series, 32 patients (86.4%) had no tumor recurrence during follow-up. Among the 5 patients who presented recurrence, 1 presented local recurrence, 1 regional recurrence, 2 locoregional recurrence, and 1 presented distant metastasis. The median time to diagnose the relapse was 15.5 months.

Of the initial treatment for patients who relapsed, 2 (40%) underwent surgical rescue with total laryngectomy, 1 (16.7%) underwent isolated radiotherapy, 1 underwent radiotherapy with rescue surgery and 1 did not undergo any treatment.

Of the 37 patients analyzed, 31 (83.8%) were alive and without disease, 3 (8.1%) had died due to the disease, and 3 had died for unrelated causes. Overall survival was 34.8 months.

## Discussion

The possibility of performing partial laryngectomy to preserve laryngeal function without compromising locoregional control of cancer is well established. It obtains the radical resection required for curative treatment, without compromising functional outcomes. Indication of partial supracricoid laryngectomy is widely accepted for the treatment of localized laryngeal carcinomas (T1b and T2) and for selected cases of advanced carcinomas (T3 and T4a),^16^ but the choice between open and transoral endoscopic procedures may be difficult due to heterogeneity lesions and patient variables to be treated.^1^

Among the characteristics of the lesions, tumor staging is one of the most important determinants of local control and survival of laryngeal cancer patients.^16^, ^19^ However, a significant number of cases may present higher pathological staging than previously defined for the primary tumor under clinical evaluation.^16^ In our study population, 5 patients were staged upwards, 2 of them from cT2 to pT3 and 2 others from cT3 to pT4a.

The correct application of indications for partial supracricoid laryngectomy allows achieving high levels of local control. In our series, local tumor control was obtained in 86% of cases, similar to that described in other retrospective studies.^1^, ^15^ However, this was associated with an organ preservation rate of 81.1%, with an overall decannulation rate of 69.3%, and an alternative permanent food route of 8.6%, indicating organ preservation outcome lower than that described in the literature.^15^ Due to the retrospective nature of the study, it is difficult to accurately assess the cause of the lower decannulation rate in relation to the literature, but some reasons can be suggested. Firstly, it is noteworthy that PHL is a procedure reserved for early laryngeal tumors (T1b and T2), but in our sample 40.5% of patients had locally advanced tumors (pT3 and pT4), which differs from findings in the literature. Studies usually demonstrate a predominantly staging of early tumors (T1a ‒ 2.8%; T1b ‒ 22.2%; T2 ‒ 72.2%; T4 ‒ 2.8%),^8^ which may partly explain lower rate of rehabilitation, as more advanced tumors often require more extensive resections and lead to greater difficulty in the recovery process of physiological activities. Another factor to take into account is the aspects of postoperative management. Laccourreye in one of his articles believes that the allocation of a non-inflated cuff tube at the end of the procedure is important to allow patients to develop the cough reflex necessary for rehabilitation,^8^ which is not routinely done in our patients. Our service is relatively new and as such presents structural problems that may have influenced this analysis. It encompassed patients who were treated from the beginning of the service’s operation to patients who were treated at the beginning of 2018. When we analyzed the average time to start speech therapy in the period 2011–2013 and compared with the period of 2016‒2018. We observe a 19-day drop in the average time to start speech therapy, which shows the maturation of the service as a whole but also exemplifies a factor that may have contributed to these rehabilitation rates found.

In our series, the frequency of salvage total laryngectomy after partial laryngectomy was 18.9%, and the main indication was rehabilitation failure. That differs from the reports in the literature that indicate local recurrence as the most common indication for salvage laryngectomy.^16^, ^20^ In two cases of cricohyodoepiglotopexy, there was anastomotic dehiscence requiring total laryngectomy, a rate higher than that reported.^15^

Thus, the major issue in the indication of partial laryngectomies remains the optimal selection of patients, especially the identification of unfavorable prognostic factors in addition to tumor size. Demographic, clinical, pathological and therapeutic characteristics may influence worse oncological and functional outcomes, as suggested by our series, and must be considered in the indication of individualized treatment.

## Conclusions

Horizontal partial laryngectomies, including supracricoid laryngectomies are an alternative to total laryngectomy with adequate local control and overall survival rates associated with maintenance of laryngeal function. On the other hand, they need specific expertise in patient selection, surgical technique and postoperative care to ensure satisfactory results.

## Declaration of competing interest

The authors declare no conflicts of interest.

## References

[1] Succo G., Crosetti E., Bertolin A. (2016). Benefits and drawbacks of open partial horizontal laryngectomies, Part A: early‐ to intermediate‐stage glottic carcinoma. Head Neck..

[2] Instituto Nacional de Câncer José Alencar Gomes da Silva (2017). Coordenação de Prevenção e Vigilância. Estimativa 2018: incidência de câncer no Brasil [Internet].

[3] Succo G., Crosetti E. (2019). Limitations and opportunities in open laryngeal organ preservation surgery: current role of OPHLs. Front Oncol..

[4] Misono S., Marmor S., Yueh B., Virnig B.A. (2014). Treatment and survival in 10,429 patients with localized laryngeal cancer: a population-based analysis. Cancer..

[5] Majer E.H., Rieder W. (1976). Technic of laryngectomy permitting the conservation of respiratory permeability (cricohyoidopexy). Ann Otolaryngol..

[6] Piquet J.J., Desaulty A., Decroix G. (1974). Crico-hyoido-epiglotto-pexy. Surgical technique and functional results. Ann Otolaryngol Chir Cervicofac..

[7] Laccourreye H., Laccourreye O., Weinstein G., Menard M., Brasnu D. (1990). Supracricoid laryngectomy with cricohyoidopexy: a partial laryngeal procedure for selected supraglottic and transglottic carcinomas. Laryngoscope..

[8] Laccourreye H., Laccourreye O., Menard M., Weinstein G., Brasnu D. (1990). Supracricoid laryngectomy with cricohyoidoepiglottopexy: a partial laryngeal procedure for glottic carcinoma. Ann Otol Rhinol Laryngol..

[9] Schindler A., Pizzorni N., Mozzanica F. (2016). Functional outcomes after supracricoid laryngectomy: what do we not know and what do we need to know?. Eur Arch Otorhinolaryngol..

[10] Park J.O., Joo Y.H., Cho K.J., Kim N.G., Kim M.S. (2011). Functional and oncologic results of extended supracricoid partial laryngectomy. Arch Otolaryngol Head Neck Surg..

[11] Zacharek M.A., Pasha R., Meleca R.J. (2001). Functional outcomes after supracricoid laryngectomy. Laryngoscope..

[12] Spriano G., Pellini R., Romano G., Muscatello L., Roselli R. (2002). Supracricoid partial laryngectomy as salvage surgery after radiation failure. Head Neck..

[13] Sewnaik A., Hakkesteegt M.M., Meeuwis C.A., De Gier H.H.W., Kerrebijn J.D.F. (2006). Supracricoid partial laryngectomy with cricohyoidoepiglottopexy for recurrent laryngeal cancer. Ann Otol Rhinol Laryngol..

[14] Nakayama M., Okamoto M., Hayakawa K. (2013). Clinical outcome of supracricoid laryngectomy with cricohyoidoepiglottopexy: radiation failure versus previously untreated patients. Auris Nasus Larynx..

[15] Thomas L., Drinnan M., Natesh B., Mehanna H., Jones T., Paleri V. (2012). Open conservation partial laryngectomy for laryngeal cancer: a systematic review of English language literature. Cancer Treat Rev..

[16] Pellini R., Pichi B., Ruscito P. (2008). Supracricoid partial laryngectomies after radiation failure: a multi‐institutional series. Head Neck..

[17] Amid M.B., Edge S., Greene F. (2018).

[18] Succo G., Peretti G., Piazza C. (2014). Open partial horizontal laryngectomies: a proposal for classification by the working committee on nomenclature of the European Laryngological Society. Eur Arch Otorhinolaryngol..

[19] De Virgilio A., Greco A., Bussu F. (2016). Salvage total laryngectomy after conservation laryngeal surgery for recurrent laryngeal squamous cell carcinoma. Acta Otorhinolaryngol Ital..

[20] Borges M.S.D., Mangilli L.D., Ferreira M.C., Celeste L.C. (2017). Apresentação de um protocolo assistencial para pacientes com distúrbios da deglutição. CoDAS..

